# Dual Barriers: Examining Digital Access and Travel Burdens to Hospital Maternity Care Access in the United States, 2020

**DOI:** 10.1111/1468-0009.12668

**Published:** 2023-08-23

**Authors:** PEIYIN HUNG, MARION GRANGER, NANSI BOGHOSSIAN, JIANI YU, SAYWARD HARRISON, JIHONG LIU, BERRY A. CAMPBELL, BO CAI, CHEN LIANG, XIAOMING LI

**Affiliations:** ^1^ University of South Carolina Arnold School of Public Health; ^2^ University of South Carolina Rural and Minority Health Research Center; ^3^ South Carolina SmartState Center for Health Care Quality University of South Carolina Arnold School of Public Health; ^4^ Division of Health Policy and Economics of the Department of Population Health Sciences Weill Cornell Medical College; ^5^ Department of Psychology University of South Carolina College of Arts and Sciences; ^6^ Department of Obstetrics and Gynecology University of South Carolina School of Medicine

**Keywords:** maternity care, rural health, digital access, access to care, telehealth, health care equity, maternal health crisis

## Abstract

**Policy Points**

- The White House Blueprint for Addressing the Maternal Health Crisis report released in June 2022 highlighted the need to enhance equitable access to maternity care.
- Nationwide hospital maternity unit closures have worsened the maternal health crisis in underserved communities, leaving many birthing people with few options and with long travel times to reach essential care.
- Ensuring equitable access to maternity care requires addressing travel burdens to care and inadequate digital access. Our findings reveal socioeconomically disadvantaged communities in the United States face dual barriers to maternity care access, as communities located farthest away from care facilities had the least digital access.

**Context:**

With the increases in nationwide hospital maternity unit closures, there is a greater need for telehealth services for the supervision, evaluation, and management of prenatal and postpartum care. However, challenges in digital access persist. We examined associations between driving time to hospital maternity units and digital access to understand whether augmenting digital access and telehealth services might help mitigate travel burdens to maternity care.

**Methods:**

This cross‐sectional study used 2020 American Hospital Association Annual Survey data for hospital maternity unit locations and 2020 American Community Survey five‐year ZIP Code Tabulation Area (ZCTA)–level estimates of household digital access to telecommunication technology and broadband. We calculated driving times of the fastest route from population‐weighted ZCTA centroids to the nearest hospital maternity unit. Rural‐urban stratified generalized median regression models were conducted to examine differences in ZCTA‐level proportions of household lacking digital access equipment (any digital device, smartphones, tablet), and lacking broadband subscriptions by spatial accessibility to maternity units.

**Findings:**

In 2020, 2,905 (16.6%) urban and 3,394 (39.5%) rural ZCTAs in the United States were located >30 minutes from the nearest hospital maternity units. Regardless of rurality, these communities farther away from a maternity unit had disproportionally lower broadband and device accessibility. Although urban communities have greater digital access to technology and broadband subscriptions compared to rural communities, disparities in the percentage of households with access to digital devices were more pronounced within urban areas, particularly between those with and without close proximity to a hospital maternity unit. Communities where nearest hospital maternity units were >30 minutes away had higher poverty and uninsurance rates than those with <15‐minute access.

**Conclusions:**

Socioeconomically disadvantaged communities face significant barriers to maternity care access, both with substantial travel burdens and inadequate digital access. To optimize maternity care access, ongoing efforts (e.g., Affordable Connectivity Program introduced in the 2021 Infrastructure Act), should bridge the gaps in digital access and target communities with substantial travel burdens to care and limited digital access.

The white house blueprint for addressing the maternal health crisis report released in June 2022 highlighted the need to enhance equitable access to maternity care.^1^ Geographic disparities in access to maternity care have been exacerbated by rural hospital‐based maternity unit closures, impeding access to maternity care for many underserved women and other pregnant, birthing, and postpartum persons.^2^, ^3^, ^4^, ^5^, ^6^ Travel distance and time can be significant barriers to accessing health care in rural areas.^7^, ^8^, ^9^ Rural birthing people often have to travel further to reach hospitals with maternity services, and this can lead to delays in care and increased rates of adverse outcomes. Perinatal care guidelines have suggested the regionalization of maternity and neonatal care within individual regions or reasonable catchment areas in such a manner that there is concentration of care for high‐risk pregnant women and their fetuses and newborns in need of high‐acuity care.^10^, ^11^ Because of the well‐documented loss of hospital‐based maternity services throughout the United States, there is increasing concern about access to maternity care, particularly in rural communities.^3^, ^8^, ^12^, ^13^

Telehealth and other innovative digital models of care delivery may hold promise for addressing some of the challenges associated with rural maternity care because of several advantages.^14^, ^15^, ^16^, ^17^ First, telehealth may be an acceptable substitute for some, but not all, in‐person prenatal and postpartum supervision care. For low‐risk pregnancies, prenatal care models typically recommend 14 visits, and utilizing telehealth as an alternative to in‐person care for some of these visits may reduce travel time and other costs for patients,^18^ including missed work hours.^19^ This is particularly important for rural residents, who often have less access to paid sick leave compared to their urban counterparts, which can amplify the financial impact of missed work hours.^20^ Second, patients may use remote patient monitoring to improve self‐efficacy of care and health outcomes. High‐risk pregnancies, in which patients have conditions like preeclampsia, hypertension, and diabetes, can use remote patient monitoring to track vital signs to help manage these clinical conditions, and remote patient monitoring may result in fewer visits with specialists.^16^, ^21^ Third, many rural communities lack obstetric care specialists such as maternal‐fetal medicine doctors and obstetricians who can provide high‐acuity care.^21^, ^22^, ^23^ Using telehealth, patients can work with their local providers to videoconference or audioconference with a specialist, allowing them access to timely and cost‐effective care.^24^, ^25^ Fourth, telehealth can improve direct communication between patients and providers. Web‐based platforms, for example, can allow birthing people to message clinicians directly for preconception, prenatal, and postpartum inquiries.^26^, ^27^ Despite these potential promises of telehealth, telehealth does not replace in‐person examinations needed for maternity care, including for ultrasounds, lab tests, and vaccinations. Furthermore, data are limited regarding how amenable telehealth is for rural families living in communities with poor access to maternity care, including the extent to which birthing persons have access to instructions and supplies to monitor critical measurements, including blood pressure, fetal heart rate, and fundal height.

Relying heavily on telehealth to meet existing gaps in in‐person maternity care without understanding patient barriers can inadvertently increase disparities in access to care. These patient barriers, including limited access to internet connectivity, digital devices, and digital literacy skills among underserved maternal individuals can disproportionately affect marginalized communities, exacerbating disparities in access to care. For example, broadband access remains lower in rural than in urban areas.^28^ In a 2021 Federal Communications Commission report, approximately 42 million Americans did not have the ability to obtain a broadband internet connection.^29^ Second, digital access to telecommunications technology is a prerequisite to benefit from telehealth.^30^, ^31^, ^32^ In rural areas, particularly small, low‐income communities, lack of technology, including video‐enabled laptops, phones, and/or tablets, and remote monitoring instruments, and/or low digital literacy further restricts service utilization.^33^ Although there is extensive information available regarding broadband availability,^34^, ^35^ little research has examined the intersection of broadband availability, digital access to devices (or its absence), and travel burdens as barriers to services utilization, making it difficult to effectively tailor telehealth policy in order to improve health care equity.

Prior research examined geographic access to maternity care units across the US counties.^4^, ^5^ However, currently, there are no studies that seek to understand the associations between travel time to hospital‐based maternity care and access to digital tools, which can be used to reach maternity underserved areas and provide tailored approaches to community needs. To address this gap, we conducted a national study to examine geographic access to hospital‐based maternity units and digital access to telecommunications technology. We examined the degree to which long driving time to hospital maternity units may be ameliorated by digital access to maternity services in urban and rural communities.

## Methods

### Study Design and Data Sources

We conducted a cross‐sectional geographic analysis at the ZIP Code Tabulation Area (ZCTA) level. Data were primarily drawn from two sources: (1) the 2020 American Hospital Association (AHA) Annual Survey, which was used to capture hospital maternity units and their locations, and (2) 2020 American Community Survey (ACS), which provided ZCTA level's 5‐year estimates of demographic characteristics and digital access to technology (any digital device, broadband subscriptions, smartphones, and tablets). Driving times in minutes from residential ZCTAs to the nearest hospital maternity units were calculated using the population‐weighted ZCTA centroid and maternity unit location based on traffic on a typical Tuesday at 9AM, using Microsoft MapQuest Application Programming Interface. We also used the 2013 Rural‐Urban Commuting Area code to define whether a community is rural or urban. The final analytic data included 14,052 (45.3%) rural ZCTAs and 17,512 (54.7%) urban ZCTAs in the United States.

The 2020 AHA Annual Surveys were used in conjunction with the Centers for Medicare and Medicaid Services Provider of Services files to identify address locations of nationwide hospital‐based maternity units (hereafter, maternity units), merging by Medicare Provider Numbers. Hospital websites were used for 18 hospitals using hospital names and location addresses in website searches for which the maternity unit status was missing in both AHA and Provider of Services (POS) data. After identifying and geocoding maternity units, we generated population‐weighted ZCTA centroids using five‐year population estimates from the ACS.

### Measures

#### Dependent Variables

We characterized the lack of digital access based on the proportions of households in a ZCTA possessing no digital devices and with no broadband subscription. The ACS considers all desktops, laptops, smartphones, tablets, and other portable wireless devices as digital devices (ACS Table S2801: types of computers and internet subscriptions). Therefore, the lack of digital access was calculated as proportions of households in a ZCTA that reported none of the aforementioned devices, ranging from 0% to 100%, with higher values signifying lower digital access. We further calculated the proportions of households that reported not having a type of telecommunication device separately: desktop/laptop, smartphone, and tablet or other portal devices. Lack of broadband subscription was calculated by the proportions of households in a ZCTA without broadband of any type: cellular data plan, cable, fiber optic or Digital Subscriber Line (DSL), and/or satellite internet services.

#### Independent Variable

Driving times from residential ZCTAs to hospital maternity units were calculated using the population‐weighted ZCTA centroid and facility location from the 2020 AHA Annual Survey using a previously documented algorithm.^4^, ^36^ A hospital maternity unit was defined if one reported all of the following services in the AHA Annual Survey: self‐reported provision of obstetric services (labor and delivery care), at least level 1 status for maternity care (that is, the provision of services for uncomplicated maternity and newborn cases), at least one dedicated obstetric bed in the hospital, and at least 10 births per year. A total of 656 hospitals with discrepancies across the four columns were complemented and validated against the Centers for Medicare and Medicaid Services POS files where obstetric services were coded as 1) no services, 2) provided by staff (i.e., hospital employed physicians), 3) provided by arrangement (e.g., physician contracts), and 4) provided both by staff and arrangements. An additional 18 hospitals with missing obstetric services status in both AHA and POS files were manually coded based on the hospital websites using hospital names and location addresses in website searches on January 5, 2021. Longitude and latitude were derived from a facility's address, and one‐way driving time (in minutes) based on the traffic density was estimated using the Microsoft MapQuest application.^37^ A cut‐off of 30 minutes was selected in accordance with the commonly cited network adequacy travel time criteria for primary care.^38^, ^39^, ^40^ Rurality of a residential ZCTA area was defined using the 2013 Rural‐Urban Commuting Area code where Rural‐Urban Commuting Area primary codes of 1–3 were considered urban and 4–10 were considered rural.

#### Covariates

ZCTA characteristics that could be associated with maternity care availability and digital access included proportions of residents that are females aged 15–44 years (i.e., of reproductive‐age), proportion of reproductive‐age women by race and ethnicity, and socioeconomic status (poverty rates, uninsured rates, unemployment rates).^5^

### Statistical Analysis

We compared rural and urban ZCTAs’ population sociodemographic characteristics and household internet and digital access by driving time to the nearest maternity unit: <15, 15 to <30, and ≥30 minutes in 2020 using Kruskal–Wallis tests. Rural vs. urban areas’ geographic access to maternity unit by driving time was compared using the two‐sided Mantel‐Haenszel χ^2^ test or Fisher's exact tests as appropriate. Rural‐urban stratified generalized median regression models were conducted to examine differences in ZCTA‐level proportions of households *lacking* digital access devices (any digital device, smartphones, and tablet) and broadband subscriptions by driving time to maternity units. The final model controlled for the aforementioned ZCTA‐level covariates and residential state.

State‐level proportions of reproductive‐age women living in ZCTAs ≥30 minutes to the nearest hospital maternity units and proportions of households without digital access were calculated separately for rural and urban communities.

We used SAS version 9.4 for statistical analyses. An institutional review board exemption was granted for this secondary data analysis. Strengthening the Reporting of Observational Studies in Epidemiology (STROBE) guidelines for cross‐sectional studies were followed.

## Results

Approximately 39.5% (*n* = 5,728) of rural ZCTAs and 16.6% (*n* = 2,905) of urban ZCTAs were ≥30 minutes from the nearest maternity unit (Table 1). Urban ZCTAs where the nearest maternity unit was ≥30 minutes away vs. <15 minutes away had higher poverty rates (median 12.0% vs. 11.2%) and both urban and rural ZCTAs ≥30 minutes away had higher uninsured rates than those <15 minutes away (rural: median 7.6% vs. 7.1%; urban: 7.0% vs. 6.6%; both *p* < 0.001).

**Table 1:** ZIP code Tabulation Areas‐level Characteristics and Digital Access to Technology in Rural and Urban Communities by One‐Way Driving Time to Nearest Hospital Maternity Units, 2020

	<15 Minutes from Maternity Units		15 to <30 Minutes from Maternity Units		30+ Minutes from Maternity Units	
	Median	IQR	Median	IQR	Median	IQR
Rural communities, *n* = 14,512	*n* = 2,562		*n* = 6,222		*n* = 5,728	
Population age and race mix, %						
Female Aged 15–44	50.5	48.9–52.1	48.7	47.2–52.0	49.6	46.3–52.3
American Indian/Alaska Native	0.2	0.0–0.9	0.0	0.0–0.5	0.0	0.0–0.8
Hispanic	3.2	1.3–7.8	1.6	0.0–4.4	1.5	0.0–5.3
Non‐Hispanic Asian	0.4	0.0–1.0	0.0	0.0–0.5	0.0	0.0–0.3
Non‐Hispanic Black	0.9	0.1–4.0	0.1	0.0–1.5	0.0	0.0–1.3
Non‐Hispanic White	93.0	84.8–96.7	96.2	90.0–98.7	95.9	87.7–99.0
Population socioeconomic status, %						
Below poverty level	13.8	9.0–19.3	11.9	7.2–18.3	13.6	7.5–20.8
Uninsured	7.1	4.3–11.2	6.8	3.7–11.9	7.6	3.8–13.4
Unemployment	4.1	2.3–6.4	3.7	1.4–6.5	3.5	0.0–7.4
Household technology access, %						
No digital device	11.5	8.0–15.2	12.2	7.8–17.6	13.6	8.0–20.5
No smartphone	23.0	18.3–27.8	24.7	18.9–31.5	27.6	20.5–36.3
No tablet/portable device	44.9	39.2–51.1	47.1	39.5–55.3	50.6	41.6–60.3
No broadband subscription	18.9	14.7–24.5	20.5	14.6–28.1	23.4	16.0–32.5
Urban communities, *n* = 17,519	*n* = 6,887		*n* = 7,727		*n* = 2,905	
Population age and race mix, %						
Female Aged 15–44	51.1	49.8–52.4	50.5	48.7–52.2	50.0	47.1–52.5
American Indian/Alaska Native	0.2	0.1–0.6	0.1	0.0–0.4	0.0	0.0–0.4
Hispanic	8.2	3.8–17.6	3.4	1.1–8.3	1.5	0.0–4.8
Non‐Hispanic Asian	2.9	1.0–6.9	0.6	0.0–2.2	0.0	0.0–0.5
Non‐Hispanic Black	6.0	2.0–16.7	1.5	0.1–7.4	0.3	0.0–4.2
Non‐Hispanic White	77.6	59.8–88.2	92.2	80.2–96.9	95.4	86.4–98.7
Population socioeconomic status, %						
Below poverty level	11.2	6.5–19.0	8.5	4.9–14.0	12.0	6.6–18.8
Uninsured	6.6	4.0–10.8	5.6	3.0–9.9	7.0	3.4–11.7
Unemployment	4.6	3.3–6.4	3.8	2.3–5.8	4.0	1.3–6.8
Household technology access, %						
No digital device	6.7	3.9–10.3	7.2	4.0–11.6	11.4	6.0–18.3
No smartphone	14.6	10.4–19.7	16.8	11.7–22.8	23.7	16.5–32.5
No tablet/portable device	36.8	29.0–45.6	37.7	29.3–46.5	46.4	37.4–56.8
No broadband subscription	12.2	7.8–18.2	13.2	8.2–19.9	20.2	12.8–29.5

Abbreviations: IQR, interquartile range of quartile 1 and quartile 3.

Notes: Driving times from residential ZIP Code Tabulation Areas to hospital maternity units were calculated using the population‐weighted ZIP Code Tabulation Area centroid from US Department of Housing and Urban Development's Office of Policy Development and Research and facility location from the 2020 American Hospital Association Annual Survey. All *p* values, which were calculated from Kruskal–Wallis tests to compare median distributions by driving time, were <0.001.

**Figure 1:**
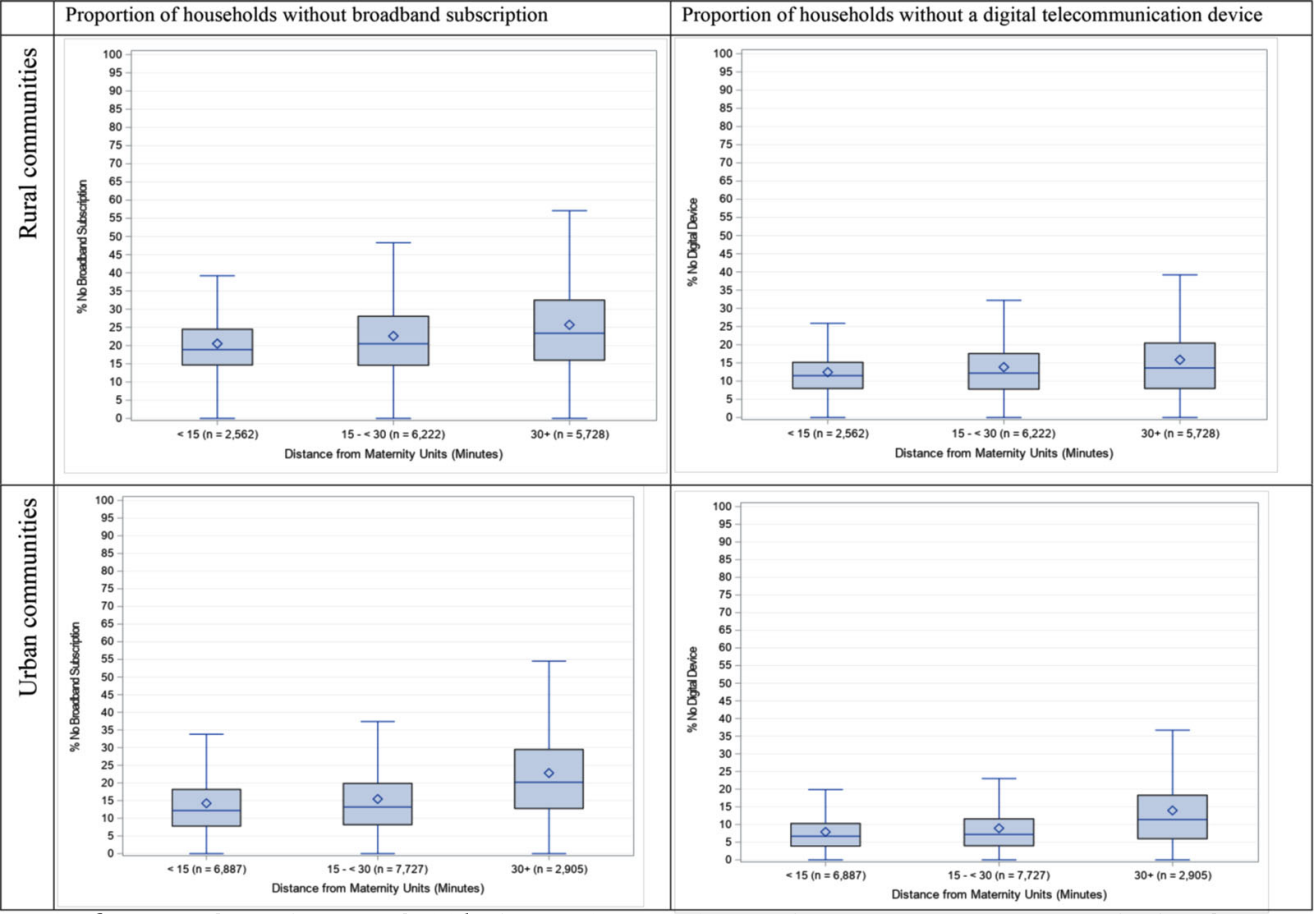
Box Plots of Rural and Urban ZIP code Tabulation Areas–Level Proportions of Households Without Broadband Subscription and Digital Device [Colour figure can be viewed at wileyonlinelibrary.com] Notes: Driving times from residential ZIP code Tabulation Areas to hospital maternity units were calculated using the population‐weighted ZIP code Tabulation Areas centroid from US Department of Housing and Urban Development's Office of Policy Development and Research and facility location from the 2020 American Hospital Association Annual Survey.

Both rural and urban communities that were ≥30 minutes from the nearest maternity unit had higher percentages of households without digital devices and broadband subscriptions than communities <15 and 15 to <30 minutes from the nearest maternity unit (Figure 1). Lack of digital access increased linearly by driving time groups from the nearest maternity unit. Specifically, median percentages of households without any digital devices were highest among both rural and urban communities ≥30 minutes from a hospital maternity unit (rural, 13.6%; urban: 11.4%), followed by communities 15 to <30 minutes from a hospital maternity unit (rural: 12.2%; urban, 7.2%), then the communities <15 minutes (rural, 11.5%; urban, 6.7%). Rural communities ≥30 minutes from the nearest maternity unit had the greatest digital access burden, surpassing urban communities with the same driving time (13.6% vs. 11.4% without digital devices, 23.4% vs. 20.2% without broadband subscription; Figure 1; 27.6% vs. 23.7% without a smartphone, 50.6% vs. 46.4% without a tablet or portable device; Appendix Figure 1).

**Figure 2:**
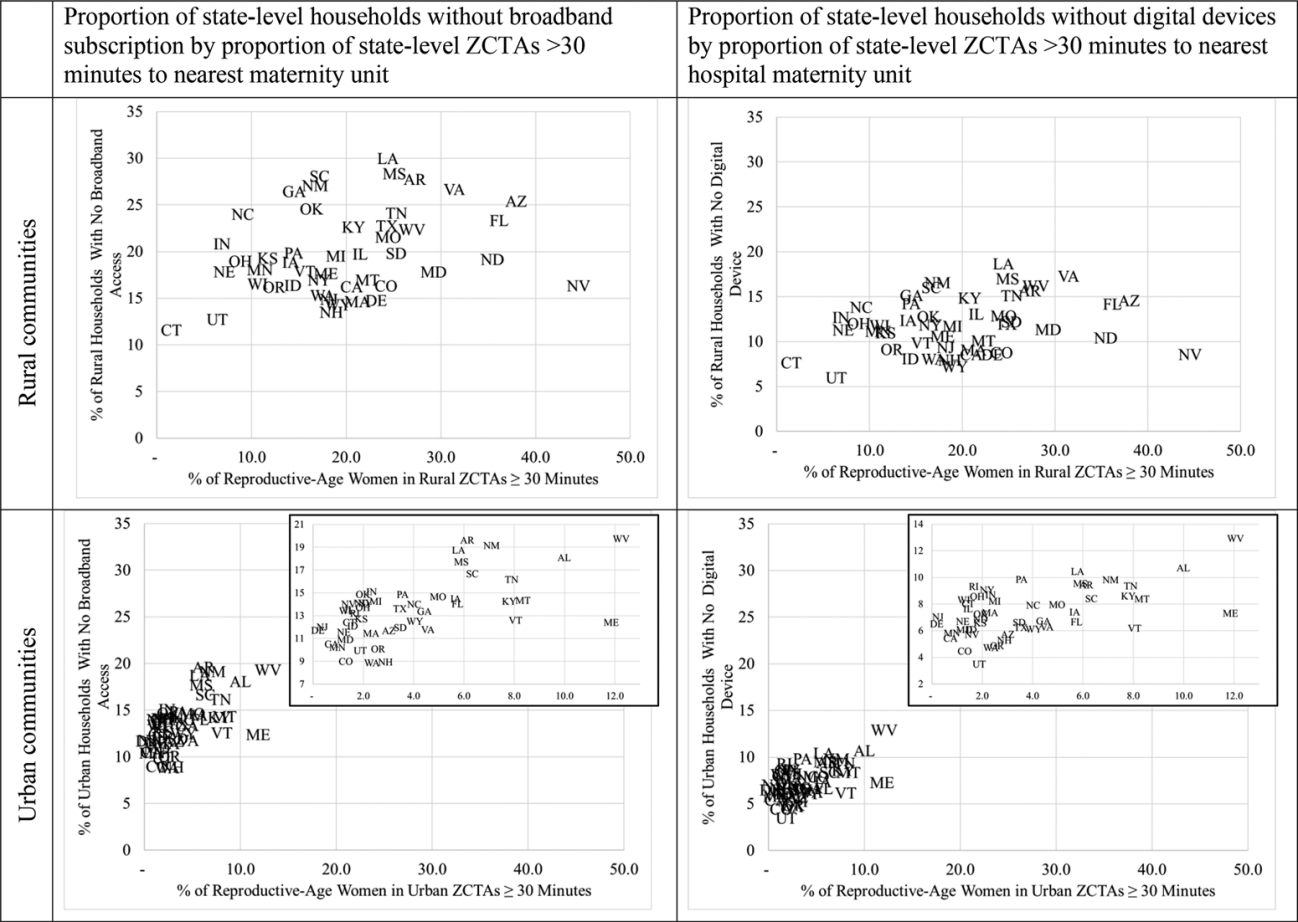
Scatterplots of State‐Level Proportions of Rural and Urban Households Without Broadband Subscription and Digital Device by Having to Drive ≥30 Minutes to Hospital Maternity Units, 2020 Notes: Data on 2020 American Hospital Association Annual Survey Data and 2020 state‐level estimates from American Community Surveys (Table S2801: types of computers and internet subscriptions) were used to visualize these state‐level variations. Abbreviation: ZCTA, ZIP Code Tabulation Area.

Figure 2 illustrates state‐level proportions of rural or urban ZCTAs ≥30 minutes from the nearest maternity facility and associated proportions that lack digital access. States with higher proportions of communities needing to drive ≥30 minutes to a maternity unit also had higher percentages of rural households lacking technology access, including any digital devices (Figure 2), broadband subscriptions (Figure 2), smartphones (Appendix Figure 2), and tablets/portable devices (Appendix Figure 2). Across states, rural communities had more variation in the proportion of communities with ≥30‐minute driving disparities, but relatively less variation in the proportion that lacked digital device access than did urban communities.

Table 2 compares percentages of households lacking digital devices and broadband by driving time to the nearest maternity unit within rural and urban communities. In both rural and urban areas, lack of digital devices and broadband subscription were significantly more prevalent in those that were ≥30 minutes vs. <15 minutes from the nearest maternity unit (Table 2). The magnitude of difference in digital access was larger in urban communities. For example, the adjusted median difference in proportions of households without broadband subscription for urban communities >30 minutes from nearest maternity unit was 5.7 (95% confidence interval 5.3–6.2), exceeding the median difference in rural communities (3.5 [3.0–4.0]) the same proximity away.

**Table 2:** Median Regression Analysis on the Associations between Physical Proximity and Digital Access to Equipment and Internets for Telehealth Services in the United States, 2020

Rural Communities					
		Driving Times to Nearest Hospital Maternity Units (Ref. <15 Minutes [*n* = 2,562 ZCTAs])			
		15 to <30 Minutes vs. <15 Minutes		30+ Minutes vs. <15 Minutes	
		Median Differences in Percentages of Households Without a Digital Device (95% Confidence Interval)	*p* Values	Median Differences in Percentages of Households Without a Digital Device (95% Confidence Interval)	*p* Values
No digital device	Unadjusted	0.7 (0.4–1.0)	<0.001	2.1 (1.7–2.5)	<0.001
	Adjusted^a^	0.9 (0.6–1.2)	<0.001	1.7 (1.4–2.0)	<0.001
No smartphone	Unadjusted	1.7 (1.2–2.2)	<0.001	4.6 (4.0–5.2)	<0.001
	Adjusted^a^	1.3 (0.9–1.8)	<0.001	3.6 (3.1–4.1)	<0.001
No tablet	Unadjusted	2.2 (1.6–2.8)	<0.001	5.7 (5.0–6.3)	<0.001
	Adjusted^a^	2.4 (1.9–2.8)	<0.001	4.5 (3.9–5.0)	<0.001
No broadband subscription	Unadjusted	1.6 (1.1–2.1)	<0.001	4.5 (4.1–4.9)	<0.001
	Adjusted^a^	1.8 (1.4–2.2)	<0.001	3.5 (3.0–4.0)	<0.001

Urban Communities					
		Driving Times to Nearest Hospital Maternity Units (Ref. <15 Minutes [*n* = 6,887 ZCTAs])			
		15 to <30 Minutes vs. <15 Minutes		30+ Minutes vs. <15 Minutes	
		Median Differences (95% Confidence Interval)	*p* Values	Median Differences (95% Confidence Interval)	*p* Values
No digital device	Unadjusted	0.5 (0.3–0.7)	<0.001	4.7 (4.3–5.1)	<0.001
	Adjusted^a^	0.5 (0.3–0.6)	<0.001	2.9 (2.5–3.2)	<0.001
No smartphone	Unadjusted	2.2 (1.9–2.5)	<0.001	9.1 (8.6–9.6)	<0.001
	Adjusted^a^	0.9 (0.6–1.1)	<0.001	5.5 (4.9–6.2)	<0.001
No tablet	Unadjusted	0.9 (0.4–1.4)	<0.001	9.6 (8.8–10.4)	<0.001
	Adjusted^a^	1.0 (0.6–1.4)	<0.001	5.9 (5.2–6.6)	<0.001
No broadband subscription	Unadjusted	1.0 (0.7–1.3)	<0.001	8.0 (7.3–8.7)	<0.001
	Adjusted^a^	1.3 (1.0–1.5)	<0.001	5.7 (5.3–6.2)	<0.001

Abbreviations: Ref, Reference group; ZCTA, ZIP Code Tabulation Area.

^a^ Adjusted models controlled for ZCTA‐level proportions of residents that are reproductive‐age (15–44) women, proportions of reproductive‐age women by race and ethnicity, poverty rate, unemployment rates, uninsured rates, and residence state.

## Discussion

Historical systemic inequities and national declines in the number of US hospital maternity units has resulted in three quarters of a million reproductive‐age women needing to travel over 30 minutes to a maternity unit in 2020.^9^, ^41^ In this study we found significant disparities in both digital access and travel burdens to accessing hospital maternity care across and within rural‐urban communities. State‐level variations also indicate structural misallocation of hospital maternity care across the system. Within states, we still found wide variations in digital access to technology, especially within urban communities. Areas farther away from the nearest hospital maternity unit had lower digital access compared to communities with proximal access to maternity units. These structural imbalances suggest that people living in underserved areas face challenges in access to the digital tools to use telehealth, and therefore bear the least digital access to timely specialty maternal care relative to their urban/rural counterparts. These areas also had higher poverty and uninsurance rates, which create additional barriers to accessing maternity care beyond travel burdens and inadequate digital access.

Travel burdens in rural America pose a significant challenge to rural health care access.^17^, ^42^, ^43^ Rural communities, especially those in frontier and/or remote areas, face persistent barriers to maintaining local maternity units,^3^, ^4^, ^5^, ^6^, ^44^ challenging the ongoing efforts to ensure access to maternity care. Several statewide studies have found that travel burdens to maternity care were associated with lower rates of prenatal care uptake, and higher rates of preterm births, low birthweights, and neonatal mortality.^45^, ^46^, ^47^ In a rural Kentucky study, longer travel distances to maternity care were found to be associated with lower prenatal care uptake. This association was most pronounced for women with lower incomes,^46^ who face additional barriers to travel such as transportation and childcare.

Nonetheless, urban communities that are >30 minutes away from maternity units also experience limited digital connectivity, making them vulnerable to the digital divide. Expanding access to telehealth maternity care is critical in these underserved areas^14^, ^48^, ^49^ where pregnant individuals often have no local access to maternity care specialists and where access to evidence‐based maternity care is challenging.^50^ Digital technology can provide access to telehealth consultations and remote perinatal support, which is especially beneficial for families living in remote or underserved areas.^48^, ^49^ Yet, despite the critical needs of underserved communities with little to no access to maternity care specialists, the current study uncovered that these residents in these communities bear the least digital access to technology. This “digital divide” in both rural and urban underserved communities is likely to exacerbate disparities in maternal health outcomes, despite technological advances and national trends toward greater use of telehealth. Understanding the current landscape of travel and digital access burdens, therefore, is pivotal to informing resource allocation and appropriately tailoring interventions to meet the needs of these underserved populations.

To address the maldistribution of hospital maternity units and digital access, the current efforts to maternity care access equity may benefit from a well‐navigated perinatal referral network incorporating both travel burden and digital accessibility to maternity care. For example, communities where a maternity unit is not easily accessible (nearest unit >30 minutes away) may be prioritized for strategies to mitigate digital access burdens, including providing video‐enabled tablets and receiving discounts for internet service bills, as well as strategies that do not rely on digital access, including expanding obstetric readiness within local primary care settings rather than relying on largely inaccessible telehealth. Furthermore, given substantial state variation in telehealth licensure policies and Medicaid coverage of video and audio‐only visits and remote monitoring,^51^ tailored strategies related to telehealth should take state‐level policies into consideration.

Spatial accessibility to maternity care is not the only important element of access to maternity care, and digital access to technology is necessary in order for these communities to benefit from telehealth expansion. Addressing barriers to maternity care access will require a multifaceted approach that considers both virtual and physical access to care. In 2019, the Federal Communications Commission (FCC) proposed the Rural Digital Opportunity Fund to improve broadband access in areas that were lacking appropriate connectivity.^29^ Driven by the success of the Connect America Fund Phase II, $20.4 billion was allocated over 10‐years to “bridge the digital divide” via a two‐phase, competitive reverse auction for providers.^52^ However, after the first two years, the FCC was criticized for failing to consider the issue of affordability.​ Thus, an Affordable Connectivity Program (ACP) was included in the 2021 Infrastructure Act, providing subsidies to help low‐income families with the cost of broadband connectivity.^53^ Our research findings highlight the need to scale up the ACP and assess its effectiveness at serving rural and underserved urban reproductive‐age households who face challenges in both physical and digital access to maternity care. In other words, these efforts should be tailored to local communities’ infrastructure to provide targeted support to communities with substantial travel burdens to care and limited digital access.

### Limitations

First, using only ZCTA‐level data pose a risk of ecological fallacy, such that assumptions about individual patients are made from aggregated data. Second, ongoing investigations are needed to assess broadband access in light of recent investments made in rural communities. The ACS data quality on digital access and sociodemographic characteristics in 2020 might be affected by the COVID‐19 pandemic because of lower response rate. To account for these disruptions, the Census Bureau implemented modifications to the ACS's weighting procedures for the 2020 data.^54^ This study relied on these weighting procedures for ZCTA‐level estimates. Third, data used for this study are limited to hospital‐based maternity units, which may have underestimated access to freestanding birth centers. Nevertheless, our data included all nationwide hospitals where over 98% of childbirths occur. Given that birth centers are an important environment for rural communities to access low‐acuity maternity care, future studies should be aimed at measuring these access points. Also, this study defined broadband subscriptions broadly, encompassing subscriptions to cellular data plans, cable, fiber optic or DSL, and/or satellite internet services. The potentially heterogeneous impact of broadband type and speed warrants further investigation. Finally, this study does not examine additional barriers to telehealth, including clinician‐level telehealth provision and patient‐level insurance coverage of telehealth services, which may modify the associations between travel time to hospital‐based maternity units and digital access burden.

## Conclusion

This study provides the first national‐level examination of both geographic access and potential digital access to hospital maternity care, and whether digital access might reduce the gaps of travel burdens across the US communities. Our findings suggest that digital access resources are less likely to be located where they are needed most: in rural America and in communities farther from hospital maternity units. Policymakers should consider not only supply‐side telehealth availability but also demand‐side digital accessibility. There is a further need to explore tailored interventions to address different resource allocation to appropriately address the maldistribution of digital access to maternity care.

*Acknowledgments*: This project was supported by the cc (NIH) (U01HD110062) and USC Big Data Health Science Center (BDHSC) under a pilot project grant (#BDHSC‐2021‐10). Its contents are solely the responsibility of the authors and do not necessarily represent the official views of the NIGMS or NIH. This information or content and conclusions are those of the authors and should not be construed as the official position or policy of, nor should any endorsements be inferred by HHS or the U.S. Government.

*Conflict of Interest Disclosures*: Nothing to disclose.

## Supporting information

Appendix Figure 1

Appendix Figure 2
