# Supplementary material for: Dual Barriers: Examining Digital Access and Travel Burdens to Hospital Maternity Care Access in the United States, 2020
Source: Milbank Q. 2023 Aug 23;101(4):1327–47. doi: 10.1111/1468-0009.12668 (PMC10726888; doi:10.1111/1468-0009.12668)
Supplement: Supplementary file 1 — Appendix Figure 1 [file MILQ-101-1327-s002.pdf]

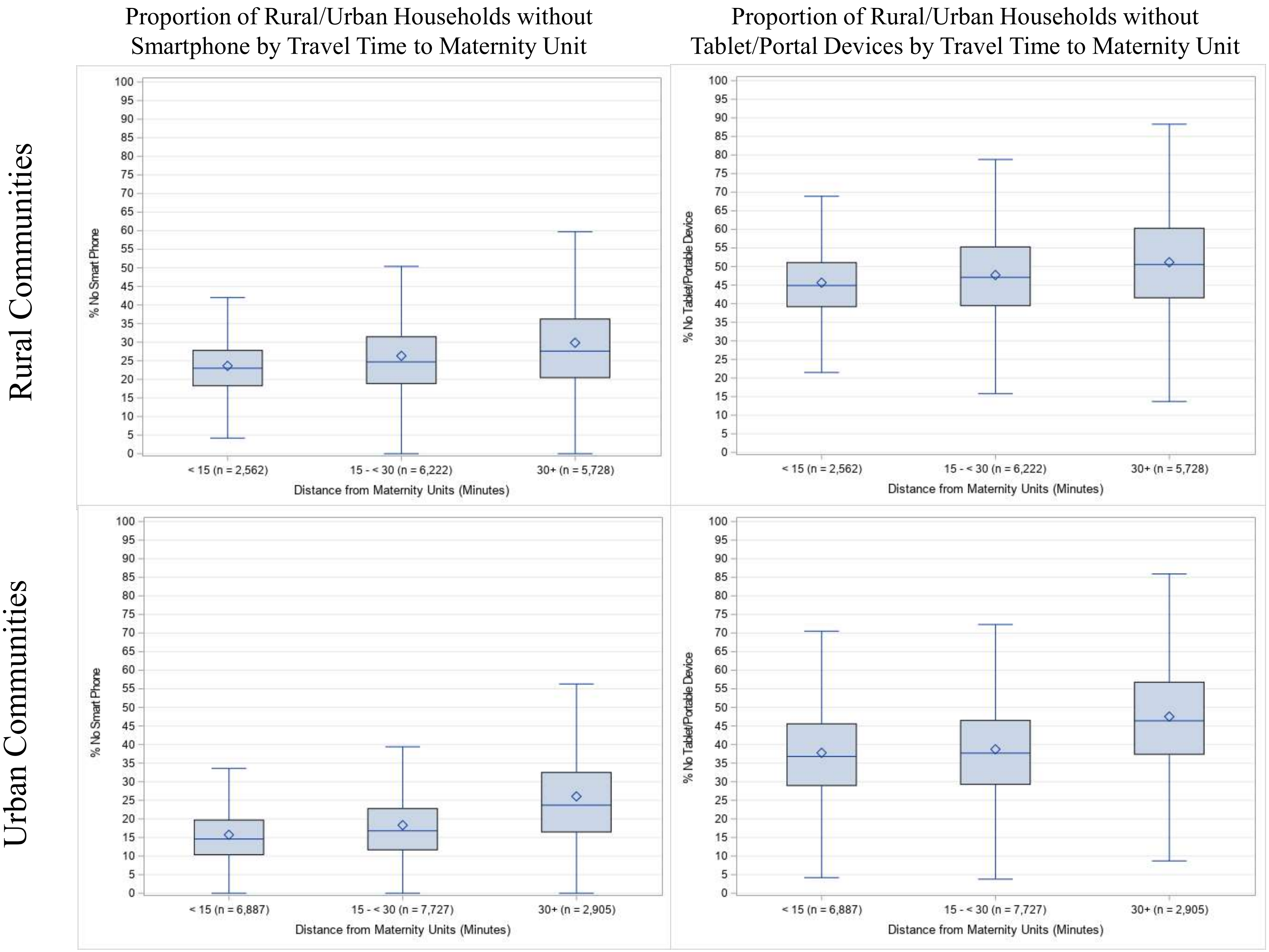
Proportion of Rural/Urban Households without Smartphone by Travel Time to Maternity Unit
Proportion of Rural/Urban Households without Tablet/Portal Devices by Travel Time to Maternity Unit
Rural Communities
Urban Communities
% No Smart Phone
% No Tablet/Portable Device
0
5
10
15
20
25
30
35
40
45
50
55
60
65
70
75
80
85
90
95
100
< 15 (n = 2,562)
15 - < 30 (n = 6,222)
30+ (n = 5,728)
< 15 (n = 6,887)
15 - < 30 (n = 7,727)
30+ (n = 2,905)
Distance from Maternity Units (Minutes)
