# Supplementary material for: Dual Barriers: Examining Digital Access and Travel Burdens to Hospital Maternity Care Access in the United States, 2020
Source: Milbank Q. 2023 Aug 23;101(4):1327–47. doi: 10.1111/1468-0009.12668 (PMC10726888; doi:10.1111/1468-0009.12668)

Proportion of State-level Rural Households without Smartphone by Proportion of Reproductive-Age Women in Rural ZCTAs >30 Minutes to Nearest Maternity Unit

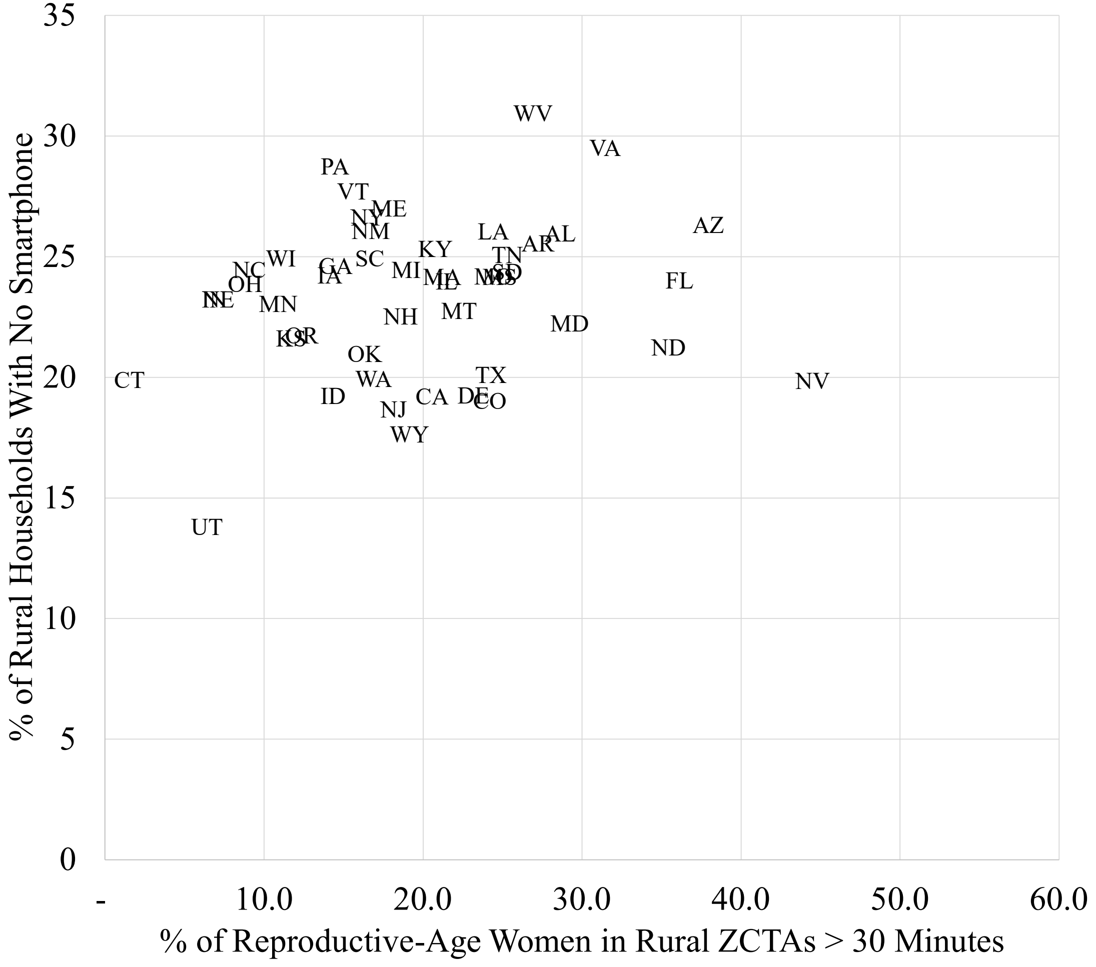

Proportion of State-level Urban Households without Smartphone by Proportion of Reproductive-Age Women in Urban ZCTAs >30 Minutes to Nearest Maternity Unit

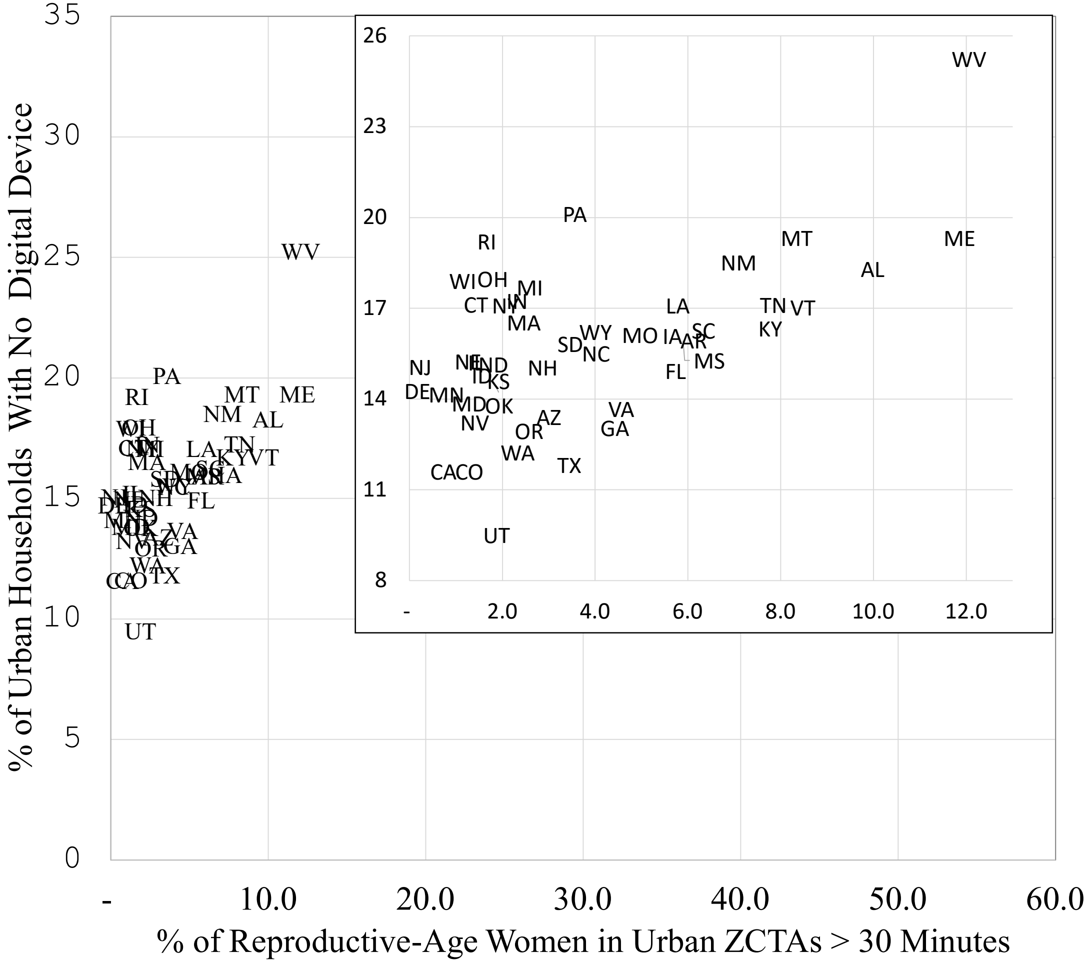

Supplement: Supplementary file 2 — Appendix Figure 2 [file MILQ-101-1327-s001.pdf]
